# Impact of HIV on Cell Survival and Antiviral Activity of Plasmacytoid Dendritic Cells

**DOI:** 10.1371/journal.pone.0000458

**Published:** 2007-05-23

**Authors:** Jennifer Hartt Meyers, J. Shawn Justement, Claire W. Hallahan, Eric T. Blair, Yongming A. Sun, M. Angeline O'Shea, Gregg Roby, Shyam Kottilil, Susan Moir, Colin M. Kovacs, Tae-Wook Chun, Anthony S. Fauci

**Affiliations:** 1 Laboratory of Immunoregulation, National Institute of Allergy and Infectious Diseases, National Institutes of Health (NIH), Bethesda, Maryland, United States of America; 2 Applied Biosystems, Foster City, California, United States of America; 3 Biostatistical Research Branch, National Institute of Allergy and Infectious Diseases, NIH, Bethesda, Maryland, United States of America; 4 Department of Medicine, University of Toronto, Toronto, Ontario, Canada; Institut Pasteur Korea, Republic of Korea

## Abstract

Plasmacytoid dendritic cells (pDCs) are important mediators of innate immunity that act mainly through secretion of interferon (IFN)-α. Previous studies have found that these cells can suppress HIV *in vitro*; additionally, pDCs have been shown to be severely reduced in the peripheral blood of HIV-infected individuals. In the present study, we sought to determine the ability of pDCs to directly suppress viral replication *ex vivo* and to delineate the potential mechanisms whereby pDCs are depleted in HIV-infected individuals. We demonstrate that activated pDCs strongly suppress HIV replication in autologous CD4^+^ T cells via a mechanism involving IFN-α as well as other antiviral factors. Of note, unstimulated pDCs from infected individuals who maintain low levels of plasma viremia without antiretroviral therapy were able to suppress HIV *ex vivo* via a mechanism requiring cell-to-cell contact. Our data also demonstrate that death of pDCs by both apoptosis and necrosis is induced by fusion of HIV with pDCs. Taken together, our data suggest that pDCs play an important role in the control of HIV replication and that high levels of viral replication *in vivo* are associated with pDC cell death via apoptosis and necrosis. Elucidation of the mechanism by which pDCs suppress HIV replication *in vivo* may have clinically relevant implications for future therapeutic strategies.

## Introduction

Dendritic cells (DCs) are bone marrow-derived cells vital to the proper development of an immune response. In humans, at least two subtypes of dendritic cells exist, myeloid DCs (mDCs) and plasmacytoid DCs (pDCs). mDCs serve as the classical antigen-presenting cells important to initiate and maintain an adaptive immune response, while pDCs are mainly linked to innate immunity [1]. Upon encountering a pathogen, pDCs produce large quantities of interferon-alpha (IFN-α), which directly inhibits viral replication and can also act to stimulate other immune competent cells [1], [2]. pDCs recognize pathogens via pattern recognition receptors, such as TLR7, which binds to viral ssRNA, and TLR9, which binds to unmethylated CpG motifs [2], [3]. Triggering of these receptors, which are located in endosomes, results in pDC activation and IFN-α production [4], [5]. Although they are capable of antigen presentation to T cells, the main functions of pDCs primarily involve type I interferon production and associated host defense early in infection.

pDCs express CD4, CXCR4, and CCR5 on their surface and have been shown to be infected by HIV [6], [7], [8], [9], [10], [11], although the *in vivo* extent and pathogenic relevance of this phenomenon remain unclear. More importantly, during primary HIV infection, numbers of pDCs and levels of IFN-α production have been shown to be severely reduced, leading to speculation that HIV disease progression may result in part from the failure of pDCs to limit viral replication [12], [13], [14], [15], [16], [17]. Upon initiation of effective antiretroviral therapy in HIV-infected individuals, the levels of pDCs and their associated IFN-α production have been shown to partially recover, but they rarely reach levels observed pre-infection [15], [16], [18], [19], [20]. It is unclear to date whether this reduction in pDCs is due to cell death associated with HIV infection or to migration from the bloodstream to tissue sites [21].

Despite a number of previous studies examining the interaction between HIV and pDCs *in vitro* [7], [8], [9], [10], [11], [22], [23], [24], [25], [26], [27], the ability of patient-derived pDCs to inhibit endogenous viral replication in autologous CD4^+^ T cells and the mechanisms whereby HIV infection results in a decrease in pDCs have not been fully elucidated. In the present study, we demonstrate the ability of activated pDCs from HIV-infected individuals to potently suppress HIV replication in autologous CD4^+^ T cells. Some of these suppressive effects were mediated by IFN-α, as demonstrated by the ability of an anti-IFN-αR antibody to partially block suppression; however, the data also suggested involvement of additional factors. Interestingly, unstimulated pDCs from infected individuals who maintained low levels of HIV plasma viremia without antiretroviral therapy suppressed viral replication in autologous CD4^+^ T cells via cell-to-cell contact. Consistent with this observation, DNA microarray analysis indicated that unstimulated pDCs from patients who have differing abilities to control HIV have distinct gene expression profiles. Finally, we demonstrated that decreased numbers of pDCs in the blood of HIV-infected individuals may be due in part to HIV-mediated cell death, as exposure of pDCs to a cell line expressing HIV induced substantial levels of apoptosis and necrosis, which was diminished by treatment with a drug that inhibits fusion of HIV with its target cell.

## Results

### pDCs in HIV-infected individuals retain HIV-suppressive functionality

The role that pDCs play in the pathogenesis of HIV is incompletely understood. pDCs have been proposed to inhibit HIV replication *in vivo*, but this activity has not been directly demonstrated. Hence, we set out to determine whether unstimulated or activated pDCs from HIV-infected individuals could directly inhibit viral replication in autologous CD4^+^ T cells and whether the degree of this inhibition related to disease status.

**Figure 1:**
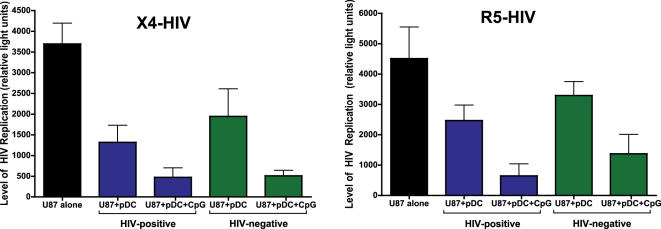
pDC-mediated suppression of HIV infection *in vitro*. U87 cell lines expressing CD4 and either CXCR4 or CCR5 were infected with pseudotyped luciferase HIV SF (X4) or HIV JRFL (R5), respectively. Purified pDCs (10^4^) from HIV-infected (n = 11) or uninfected (n = 4) individuals were cocultured with 2×10^4^ U87 cells, with or without 1 µg/ml CpG ODN 2216 in triplicate wells. After two days of incubation, cells were lysed and luciferase activity following addition of substrates was quantified by a luminometer. Error bars indicate the standard error of the mean.

We first addressed the relative abilities of pDCs from HIV-infected vs. uninfected individuals to suppress HIV replication *in vitro*. U87 cell lines expressing CD4 and either CCR5 or CXCR4 (U87-R5 or U87-X4, respectively) were infected with the appropriate R5- or X4-tropic luciferase reporter HIV, and pDCs that had been purified from either HIV-infected patients or uninfected controls were added. The level of HIV transcription was measured by luciferase activity, and the ability of CpG-stimulated or unstimulated pDCs to suppress HIV replication was quantified. Unstimulated pDCs could generally suppress HIV to a modest degree, while pDCs stimulated via TLR9 by CpG strongly suppressed virus in all instances. Of note, pDCs from HIV-infected versus uninfected individuals mediated similar levels of viral suppression (Figure 1). These data indicate that, despite the relatively low numbers of pDCs in the peripheral blood of HIV-infected individuals *in vivo*, the remaining pDCs seem to retain their antiviral activity.

### Direct suppression of endogenous HIV replication by pDCs from HIV-infected individuals

In order to analyze the capacity of patient-derived pDCs to inhibit HIV in a more physiologic setting, we conducted HIV p24 release assays in which pDCs from an HIV-infected individual were co-cultured with autologous activated CD4^+^ T cells. The degree of HIV replication was measured by HIV p24 ELISA and the level of IFN-α production by ELISA at days 3, 5, and 7. In the case of individuals receiving effective antiretroviral therapy (ART), we co-cultured pDCs from the current aviremic time point with autologous CD4^+^ T cells cryopreserved at a time when these individuals had detectable plasma viremia.

**Figure 2:**
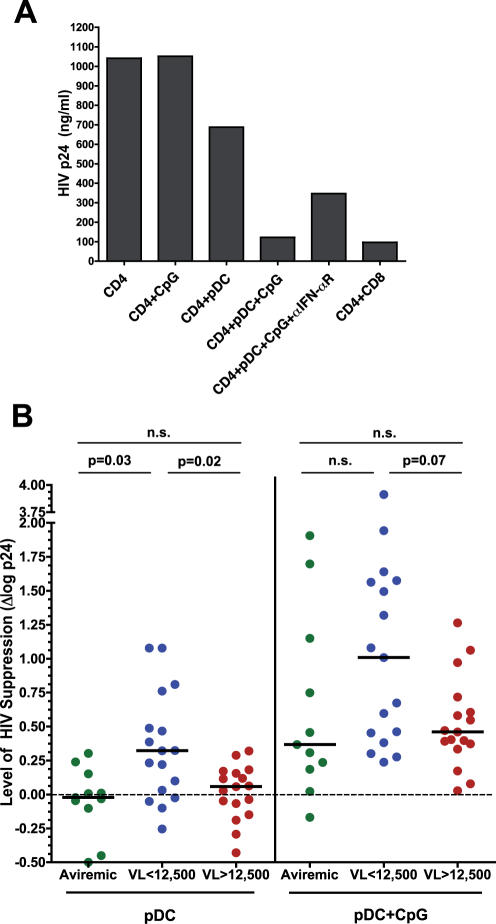
pDC-mediated suppression of HIV replication in autologous CD4^+^ T cells. pDCs were purified from HIV-infected individuals and plated overnight at 5×10^4^ cells/well with or without 1 µg/ml CpG ODN 2216. For targets, autologous PBMCs were stimulated overnight with anti-CD3 and IL-2. On day 0, CD4^+^ T cells were purified and plated at 1.2×10^6^ cells/well alone or with 5×10^4^ stimulated or unstimulated pDCs, or with 1.2×10^5^ purified autologous CD8^+^ T cells. Neutralizing antibody against IFN-α receptor was added at 5 µg/ml to some wells. Culture supernatants were collected at days 3, 5, and 7 and assayed for HIV p24 by ELISA. All data shown are from day 7. A) A representative example of data from a viremic patient. CpG ODN 2216 had no effect on CD4^+^ T cells alone, and the degree of viral suppression by activated pDCs was comparable to that by CD8^+^ T cells. B) Effect of pDCs on HIV replication in autologous CD4^+^ T cells. The degree of HIV suppression by pDCs was expressed as the difference of log HIV p24 production between the culture containing CD4^+^ T cells alone and the co-culture of CD4^+^ T cells and pDCs (Δlog p24 = log HIV p24[CD4 cells alone]-log HIV p24[CD4+pDC]). Data are shown for unstimulated pDCs (left panel) and CpG-stimulated pDCs (right panel). Horizontal lines represent median values. n.s., not significant.

In the setting of pDC-CD4^+^ T cell co-culture, CpG-stimulated pDCs strongly suppressed endogenous viral production (Figure 2). This suppression could be partially reversed by blocking the IFN-α receptor, indicating that some, but not all, of the suppression was mediated by IFN-α (Figures 2A and 4A). These data suggest that additional pDC-derived suppressive factors, possibly including IFN-α related proteins, might also be involved in the suppression of HIV replication. To further delineate the ability of pDCs from individuals at different stages of disease to inhibit viral replication, we divided the study participants into three groups, aviremic (ART-treated, plasma viremia<50 copies of HIV RNA per ml plasma), “low-viremic” (ART-naïve, plasma viremia<12,500 copies/ml, median 7,320, range 1,025–12,028), and “high-viremic” (ART-naive, plasma viremia>12,500 copies/ml, median 49,689, range 12,904–134,354). The break point of plasma viremia, 12,500, was chosen based on the median value of all ART-naïve infected individuals (12,466 copies/ml). The majority of individuals classified as low-viremic have not progressed virologically over the time they have been observed (from 1–5 years), whereas those classified as high-viremic have have been unable to control their viral load over time. Of note, although pDCs that were not stimulated with CpG were generally inefficient in suppressing HIV replication, unstimulated pDCs from the ART-naïve “low viremic” group suppressed HIV to a significantly greater degree than did those from aviremic or high-viremic individuals (p = 0.03 and p = 0.02, respectively, Figure 2B). CpG-stimulated pDCs from these low-viremic individuals also tended to suppress HIV to a greater degree than did stimulated pDCs from the other two patient groups, but the difference in suppressive capacity did not reach the level of significance (Figure 2B, right panel). These data suggest that pDCs from individuals who maintain low levels of plasma viremia have a greater antiviral capacity than those from individuals with high levels of plasma viremia. When unstimulated pDCs from uninfected individuals were cultured with CD4^+^ T cells derived from viremic individuals, their antiviral activities tended to be similar to those from high-viremic and aviremic individuals (data not shown), suggesting that pDCs from low-viremic individuals may be qualitatively different from pDCs in HIV-uninfected individuals.

**Figure 3 pone-0000458-g003:**
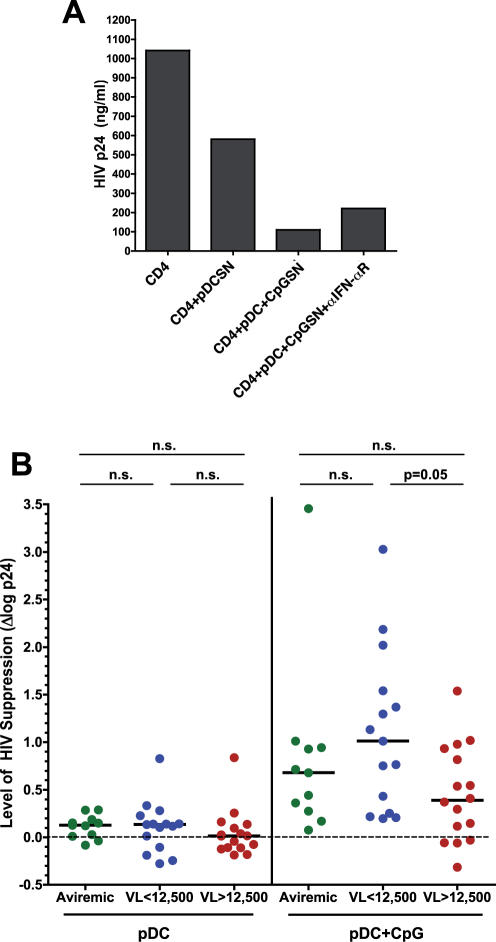
Soluble factor-mediated suppression of HIV replication. HIV p24 assays were set up as in Figure 2, with the exception that pDCs were cultured at 5×10^4^ cells/well separately from CD4^+^ T cells, and supernatants from unstimulated or CpG ODN 2216-stimulated pDC cultures were added to the T cells at days 0, 3, and 5. Data from day 7 are shown. A) A representative example of data from a viremic patient. B) Effect of soluble factors secreted by pDCs on HIV replication in autologous CD4^+^ T cells, expressed as in Figure 2. Data are shown for supernatants from unstimulated pDCs (left panel) and CpG-stimulated pDCs (right panel). Data are expressed as delta log p24 as described above. n.s., not significant.

**Figure 4:**
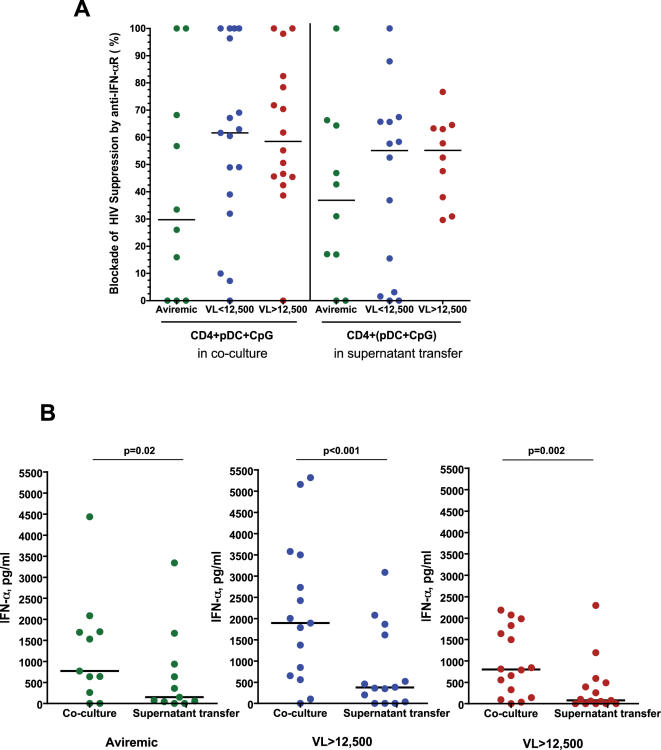
Interferon-α in pDC-mediated HIV suppression. A) Effect of neutralizing IFN-α receptor antibody on HIV suppression. The percent of the HIV suppression observed in co-culture experiments (left panel, data from Figure 2B) or pDC supernatant transfer experiments (right panel, data from Figure 3B) that could be neutralized by an antibody to the IFN-α receptor is indicated. No significant differences were observed between the degree of blockade of suppression between patient groups. B) Quantification of IFN-α production in pDC cultures. Culture supernatants from day 3 cultures of CD4^+^ T cells plus: activated pDCs (left panel of each plot, described in Figure 2) or supernatants of activated pDCs (right panel of each plot, described in Figure 3) were analyzed for IFN-α production by ELISA. Significantly more IFN-α was present in culture supernatants from CD4^+^ T cell-pDC co-culture experiments than in those from cultures containing pDC supernatants (p values indicated for each patient group).

In order to measure the soluble factor-mediated antiviral capacity of pDCs from HIV-infected individuals, we added supernatants from CpG-stimulated or unstimulated pDCs to autologous activated CD4^+^ T cell cultures. Supernatants from stimulated pDCs effectively suppressed HIV replication, and varying proportions of this suppression could be blocked by anti-IFN-αR antibody (Figures 3 and 4A). Similarly to the co-culture experiments, soluble factors produced by activated pDCs from low-viremic individuals suppressed HIV to a greater extent than did those from high-viremic individuals (Figure 3B; p = 0.05). However, no significant inhibition of HIV replication was observed with supernatants from any group of unstimulated pDCs (Figure 3B), suggesting that the suppression depicted in Figure 2B by unactivated pDCs was exerted mainly through a cell-mediated mechanism unlikely to be mediated solely by IFN-α. These data suggest that pDCs from low-viremic infected individuals may control HIV replication through a previously unexplored mechanism.

When the levels of IFN-α in culture supernatants were measured, CpG-stimulated pDCs co-cultured with autologous CD4^+^ T cells from all three groups of HIV-infected individuals were found to produce modest to high levels of this cytokine (Figure 4B). This observation was consistent with the ability of all of these cells to suppress HIV, which could be partially blocked by anti-IFN-αR. Although co-cultures containing activated pDCs from low-viremic individuals appeared to produce more IFN-α than those containing pDCs from the other two patient groups (Figure 4B), this apparent difference was not statistically significant. The neutralizing anti-IFN-αR antibody blocked HIV suppression to a widely variable and not significantly different degree in all three patient groups (Figure 4A), in agreement with their comparable levels of IFN-α production. No detectable IFN-α was produced by CD4^+^ T cells alone, indicating that pDCs were the sole source of the cytokine (data not shown). Interestingly, significantly higher levels of IFN-α were produced by activated pDCs co-cultured with CD4^+^ T cells than by activated pDCs cultured alone in the supernatant transfer experiments in all three patient groups (p≤0.02 in all cases, Figure 4B), suggesting that CD4^+^ T cell-pDC contact enhanced IFN-α production. However, there were no significant differences in IFN-α production between cultures containing unstimulated pDCs from individuals with different levels of HIV viremia (data not shown), which in most cases was below the assay's limit of detection. These data support the idea that the HIV suppression mediated by unstimulated pDCs from individuals maintaining a low viral load cannot be explained by IFN-α activity, and that an important cell contact-mediated interaction occurs between pDCs and CD4^+^ T cells.

### pDCs from individuals maintaining differing levels of viremia show distinct gene expression profiles

**Figure 5:**
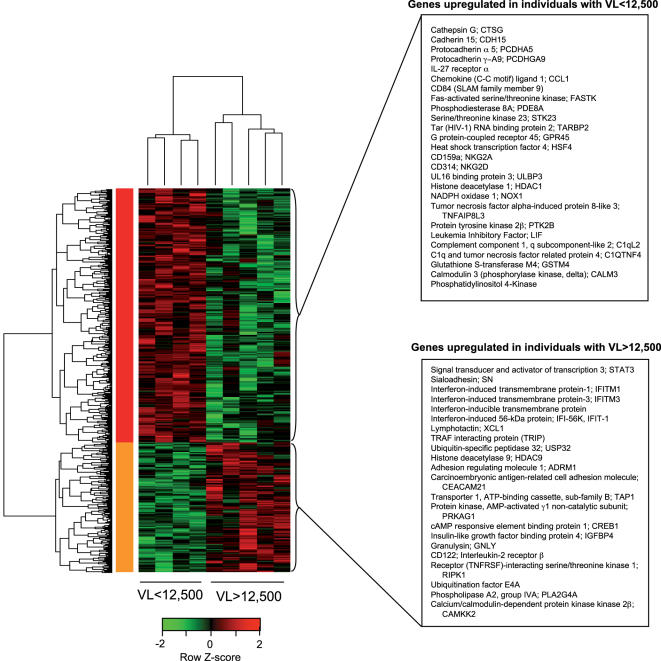
Clustering of differentially expressed genes in unstimulated pDCs from HIV-infected individuals with low versus high viral loads. cRNA was prepared from up to 10^6^ pDCs purified from 4 individuals maintaining plasma viremia<12,500 copies/mL and 5 individuals with uncontrolled viremia>12,500 copies/ml. Transcriptional profiles were assessed using Applied Biosystems high-density genome survey microarrays. Overall, 596 probes were found to be differentially expressed with p<0.05, of which examples are listed at right. Variations in relative levels of gene expression (Z-score, scale shown below heatmap) are indicated in color, where red regions indicate upregulation, and green regions indicate downregulation. The hierarchical clustering heatmap was generated using Euclidean distance as the distance measurement between clusters. All genes differentially expressed with p<0.05 are shown in supplemental Table S1.

To address whether pDCs from low-viremic individuals had a transcriptional profile distinct from that of pDCs from high-viremic individuals, we conducted DNA microarray analysis of fresh *ex vivo* pDCs. Examination of microarray data revealed that the pDCs isolated from these two groups of HIV-infected individuals clearly demonstrated distinct patterns of gene expression (Figure 5). Upon comparing the genes that were significantly more highly expressed in pDCs from low-viremic individuals versus those in pDCs from high-viremic individuals (p<0.05), we found that a large number of these genes were uncharacterized (data not shown), suggesting that the mechanism by which pDCs from low-viremic individuals suppress HIV may be mediated through genes that have been previously undescribed. Although the differences in gene expression between these two groups of pDCs were generally subtle, genes upregulated in pDCs from low-viremic individuals included those encoding adhesion molecules, molecules involved in apoptotic pathways, and several molecules that have been previously implicated in HIV suppression, including LIF and the IL-27 receptor (Figure 5) [28], [29]. The identification of a distinct gene expression profile in pDCs from low-viremic individuals versus pDCs from high-viremic individuals supports the idea that these cells may behave differently during HIV pathogenesis, particularly in their ability to suppress HIV replication.

### HIV can directly kill pDCs

**Figure 6:**
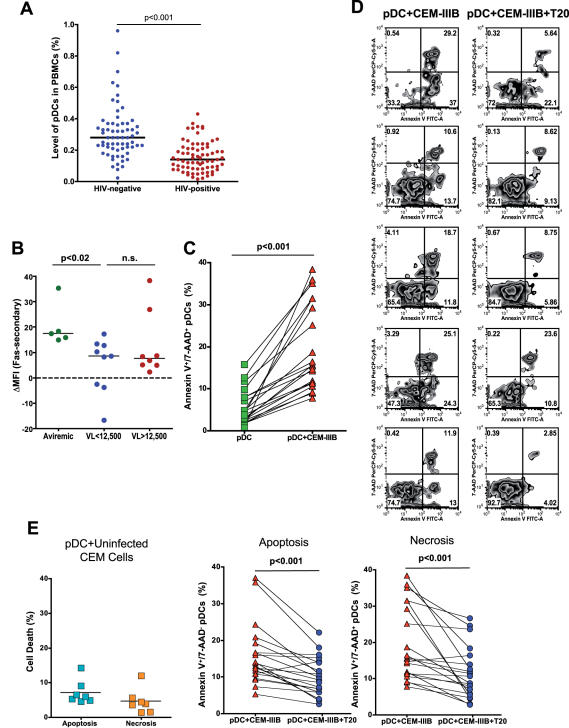
pDC killing in HIV-infected individuals. **A)** Plasmacytoid dendritic cell numbers are reduced in HIV-infected versus uninfected individuals. PBMCs were stained with anti-BDCA-2 (FITC) and anti-CD123 (PE) to measure the levels of pDCs. Horizontal lines represent median values. **B)** pDCs are susceptible to Fas-mediated apoptosis. Fas was crosslinked on the surface of PBMCs, and resultant pDC apoptosis was measured by Annexin V staining. n.s., not significant. **C–E)** pDC cell death induced by HIV-infected cells. 2×10^6^ CEM cells chronically infected with HIV (CEM-IIIB) were incubated for 8 h with 10^7^ PBMCs from HIV-infected donors. The levels of apoptosis and necrosis were assessed by Annexin V and 7-AAD staining. Cells within the pDC (BDCA-2^+^/CD123^+^) population that were double-positive for both Annexin V and 7-AAD were considered necrotic, while those positive for Annexin V and negative for 7-AAD were considered apoptotic. In some cultures, 100 µg/ml T20 was added. **C)** Cells expressing HIV induce pDC death. The percent of pDCs that were necrotic (Annexin V^+^/7-AAD^+^) is indicated on the y-axis. PBMCs alone versus PBMCs+CEM-IIIB are shown. As indicated, CEM-IIIB cells showed a significant induction of cell death vs. cells alone. **D)** Representative examples of T20-mediated blockade of CEM-IIIB-induced pDC cell death. **E)** Cell death is blocked by T20. Cells were incubated with CEM cells alone (left panel), CEM-IIIB, or CEM-IIIB+T20, and the percentage of pDCs in the apoptotic or necrotic quadrants was quantified. No cell death above baseline was induced by CEM cells alone. Both types of cell death induced by CEM-IIB cells were significantly reduced (p values indicated) in the presence of T20.

Our data thus far demonstrate that pDCs from HIV-infected individuals can suppress HIV replication; however, pDC numbers in peripheral blood are known to be decreased in HIV-positive compared to HIV-negative individuals, indicating that a loss of these cells may interfere with their ability to successfully suppress HIV *in vivo*. It has been unclear whether this reduction in pDCs is due to cell death or redistribution of pDCs into various tissue sites [21]. In order to address this issue in the context of pDC failure to contain *in vivo* HIV replication, we sought to determine whether HIV is involved in the direct killing of pDCs. First, we measured the levels of pDCs present in blood from HIV-infected and healthy volunteers by flow cytometry to confirm the reduction in cell numbers predicted by previous studies [21]. As shown in Figure 6A, HIV-infected individuals had significantly lower numbers of pDCs in peripheral blood than did HIV-negative individuals (p<0.001). We next analyzed whether pDCs were susceptible to apoptosis assessed by Annexin V and 7-AAD staining and found that pDCs from both infected and uninfected individuals underwent apoptosis when triggered via Fas/Fas-L (data not shown). However, pDCs from low-viremic individuals were significantly less susceptible to Fas-mediated apoptosis than were those from aviremic individuals (p = 0.02, Figure 6B). It could thus be postulated that pDCs from low-viremic individuals might be relatively resistant to Fas-mediated cell death *in vivo*.

In order to investigate whether decreased levels of pDCs in HIV-infected individuals could result from accelerated cell death in the setting of HIV infection, we incubated PBMCs with either recombinant HIV envelope protein gp160 (Protein Sciences) or with a CEM cell line chronically infected with HIV (CEM-IIIB) and assessed resultant pDC viability. Although no significant cell death was induced by HIV Env (data not shown), CEM-IIIB induced profound pDC death, as measured by 7-AAD and Annexin V staining (Figure 6C). Cells staining with Annexin V alone were considered apoptotic, whereas those that were stained with both Annexin V and 7-AAD were considered necrotic. The cell death, both apoptosis and necrosis, was effectively blocked by addition of the HIV fusion inhibitor T-20 (Figure 6D–E), suggesting that pDCs are killed by a mechanism involving HIV-pDC fusion. Uninfected CEM cells did not induce appreciable pDC death in this system (Figure 6E, left panel). These data suggest that the reduction in pDC numbers observed in HIV-infected individuals could be due at least in part to cell death induced by fusion of pDCs with HIV-infected cells.

## Discussion

HIV infection is associated with numerous immunologic deficiencies in nearly every type of immune competent cell, including those that are programmed to inhibit HIV replication [30], [31], [32]. A more complete understanding of the mechanisms by which HIV disrupts the antiviral properties of immune system cells could lead to new approaches to treatment and containment of HIV replication. pDCs are known to play an important role in innate immunity against viral infections by secreting IFN-α. Previous studies have demonstrated that the levels of pDCs in the peripheral blood are severely compromised in HIV-infected individuals, but the cause of this depletion, as well as the potential consequences of interactions between pDC and HIV-infected CD4^+^ T cells, remain unclear. In the present study, we have examined the abilities of pDCs from HIV-infected individuals to suppress HIV replication in autologous CD4^+^ T cells. Our data demonstrated that pDCs from infected individuals who were able to maintain a low viral load without antiretroviral therapy manifested considerable HIV-suppressive activity. As we did not observe similar antiviral activities by unstimulated pDCs from HIV-negative, aviremic, or high-viremic individuals, it is possible that pDCs require optimal stimulation *in vivo* by a low level of virus to develop potent suppressive activity, or that individuals able to control viremia have innately distinct pDCs. In support of this possible requirement for low-level stimulation by virus, previous studies have shown that HIV can induce activation and maturation of pDCs [24], [26], [27]. Activated pDCs from all patient groups studied could strongly suppress HIV via a mechanism at least partially mediated by soluble factors, including IFN-α. In addition, we demonstrated that HIV can be directly involved in the killing of pDCs and thus prevent its own suppression, suggesting that the depletion of pDCs in the peripheral blood could be in part due to HIV-mediated cell death rather than migration to tissue sites.

Previous *in vitro* studies have demonstrated that pDCs can suppress HIV replication in various cell line systems [25], [27]; however, our data provide a physiologic context for these prior observations by directly addressing the abilities of pDCs from HIV-infected individuals to suppress endogenous viral replication. The enhanced HIV-suppressive activities of pDCs from therapy-naïve infected individuals who maintain a low level of plasma viremia suggest that pDCs may play a role in the ability of these individuals to control HIV replication. A further understanding of the mechanisms by which these pDCs may suppress HIV replication *in vivo*, could provide novel targets and approaches for future therapeutic strategies.

Whereas previous *in vitro* work on pDCs obtained from HIV-infected individuals proposed that IFN-α was the major mechanism of viral inhibition [21], [33], our data suggest that pDCs can also suppress HIV via other mechanisms, involving both cell-to-cell contact and soluble factors. Signaling initiated by IFN-α can induce a number of genes [34], many of whose products are to date uncharacterized; therefore, it is possible that the cell contact-mediated mechanism involves an IFN-α stimulated transmembrane protein. However, DNA microarray analysis revealed that several IFN-stimulated genes were downregulated in pDCs from low-viremic individuals (Figure 5), which suggests that the cell-to-cell contact-mediated mechanism is likely to involve either a novel IFN-stimulated molecule or a mechanism not mediated by IFN-α. In support of this observation, a previous study demonstrated that HIV infection caused the upregulation of a number of IFN-stimulated genes whose expression did not correlate with control of HIV replication, leading the authors to conclude that some IFN-α-induced genes were ineffective in viral suppression [30]. Our microarray analysis also identified a distinct gene expression profile in pDCs from low-viremic individuals versus pDCs from high-viremic individuals, supporting the idea that pDCs from individuals with differing disease status may behave differently, both in terms of effector functions and survival. Future analysis of these gene expression profiles will be useful to delineate the mechanism of the pDC-CD4^+^ T cell contact-mediated suppression of HIV. pDC-CD4^+^ T cell contact, in addition to mediating HIV suppression in low-viremic individuals, also appeared to increase the amount of IFN-α produced by stimulated pDCs from all three groups of HIV-infected individuals. Elucidation of the mechanism by which cellular contacts between pDCs and CD4^+^ T cells enhance antiviral activities of pDCs awaits future analysis.

Finally, a decrease in peripheral blood pDCs has been shown to be associated with HIV infection, and previous studies have indicated that HIV-mediated pDC activation leads to the upregulation of CCR7 and the production of CCL2, CCL3, and CCL4 [23], [24], [26], which may lead to migration of pDCs to various tissue compartments. However, our data suggest that HIV-infected cells may also directly fuse with and kill pDCs, which could account for much of the depletion observed in infected individuals. . It has been demonstrated that HIV-mediated activation of pDCs involves endocytosis and not membrane fusion [22], so it is possible that HIV kills pDCs by a mechanism independent of that which induces their activation. These observations may have clinically relevant implications, as strategies to prevent HIV-mediated pDC death using an agent that blocks virus-cell fusion could preserve these cells and as a result enhance the innate ability of the host to suppress HIV replication.

## Materials and Methods

### Study subjects

45 HIV-infected individuals were studied for p24 assays (Figures 2–4, median viral load 9,189 copies/ml of plasma, range<50–134,354 copies/ml; median CD4^+^ T cell count per mm^3^ blood 550, range 251–1,117; Table 1). Several additional HIV-infected individuals (1 aviremic and 5 viremic) were included in the apoptosis and microarray assays. Within the limits of our period of observation, those patients classified as “low-viremic” generally did not progress virologically, whereas those classified as “high-viremic” were generally unable to control their plasma viremia. Leukapheresis was conducted in accordance with protocols approved by the Institutional Review Boards of the National Institute of Allergy and Infectious Diseases, National Institutes of Health and the University of Toronto, Ontario, Canada.

**Table 1:** Patient Characteristics

Group	n =	Median Plasma Viremia^A^ (copies/ml); [Range]	Median CD4 count (cells/mm^3^); [Range]	Antiretroviral treatment
Aviremic	11	<50; [N/A]	577; [229–1,117]	Yes
Viremia<12,500	17	7,320; [1,025–12,028]	536; [251–815]	No
Viremia>12,500	17	49,689; [12,904–134,354]	520; [270–960]	No

A Measured by ultrasensitive bDNA or RT-PCR assay with a detection limit of 50 copies per ml of plasma.

### Cell Culture Media

All cells were cultured in RPMI 1640 (Invitrogen) supplemented with 10% FCS, Penicillin/Streptomycin, and HEPES (all from Invitrogen).

### Purification of pDCs

PBMCs were isolated from leukaphereses by Ficoll-Hypaque (MP Biomedicals) density gradient centrifugation. Cells (0.8–2×10^9^) were resuspended in MACS buffer (PBS, 2 mM EDTA, 0.5% BSA) and incubated for 10 min. at 4°C with 50 µl FcR blocking reagent (Miltenyi) per 10^8^ cells. Anti-BDCA-4 beads (Miltenyi) were then added at 50 µl per 10^8^ cells followed by incubation for 15 min. at 4°C. Cells were then washed, resuspended at 2×10^8^ cells/ml, and run over an LS column following the manufacturer's protocol (Miltenyi). The eluted positively selected cells were spun down and run over an MS column following the manufacturer's protocol (Miltenyi). Cells eluted from the MS column were incubated 10 min. at 4°C with 10–20 µl anti-CD14 Dynabeads (Invitrogen) to deplete residual monocytes, which were removed via magnetic separation. The remaining enriched pDCs (∼90% pure) were then enumerated and plated at 5×10^4^ cells/well in round-bottom 96-well plates with or without stimulation with 1 µg/ml CpG (ODN 2216 [35], Operon). Antibodies used to measure pDC purity were BDCA-2-FITC (clone AC144, Miltenyi) and CD123-PE (clone 9F5, BD Biosciences).

### Luciferase HIV assays

U87 cells stably transfected with CD4 and either CCR5 or CXCR4 [36] were seeded in 50 µl at 2×10^4^ cells/well in 96-well flat-bottom plates. Cells were infected with 10 µl of luciferase HIV of the appropriate tropism (JRFL-luc [titer 1120 ng/ml] or SF33-luc [titer 900 ng/ml], respectively). The plasmids were originally obtained through the AIDS Reference and Reagent Program, Division of AIDS, NIAID, NIH, and JRFL-env was originally from Nathaniel Landau[37]. 1×10^4^ unstimulated or CpG-stimulated pDCs from either HIV-infected or –uninfected individuals were added in 50 µl to some wells. All conditions were performed in triplicate. Plates were incubated for 48 h at 37°C, after which the cells were lysed by addition of Cell Culture Lysis Reagent (Promega), frozen at −80°C, and read using the Promega Luciferase Assay detection system in a luminometer (Bio Rad Laboratories).

### HIV p24 assays

2–4×10^8^ total PBMCs from HIV-infected individuals were stimulated overnight with anti-CD3 antibody and rIL-2 (Roche). CD4^+^ and CD8^+^ T cells were then purified by negative selection using a column-based separation technique (StemCell Technologies) as previously described [38]. 1.2×10^6^ CD4^+^ T cells/mL were plated in 48-well plates on day 0, and 5×10^4^ pDCs (purified and plated as described above, then incubated overnight) with or without 1 µg/ml CpG ODN 2216 and 5 µg/ml anti-IFN-αR (Calbiochem) were added to appropriate wells. Additionally, 200 µl supernatant from pDC cultures was added to the CD4^+^ T cells in some experiments. 0.5 ml culture supernatant was collected on days 3, 5, and 7, and levels of HIV p24 and IFN-α were measured by ELISA (Beckman Coulter and PBL, respectively). All conditions were set up in single wells, and ELISA measurements were performed in duplicate. The difference in log p24 values was used as the measure of suppression.

### Microarray Analysis

RNA was prepared from up to 10^6^ pDCs using an RNeasy Micro Kit (Qiagen). 300 ng RNA was used to synthesize cRNA using a NanoAmp™ RT-IVT Labeling Kit (Applied Biosystems) followed by hybridization to Human Genome v2.0 Microarrays (Applied Biosystems) per manufacturer's protocols. DNA microarrays were washed as described and read using an Applied Biosystems 1700 Chemiluminescent Microarray Analyzer. Data normalization was then performed using quantile normalization [39]. Statistical analysis was performed using two-sample t test after filtering out probes not expressed in both conditions. A probe with signal-noise ratio (S/N) below 3 was considered not expressed for each sample, and a probe was considered not expressed for each condition if the probe was not expressed in more than half the samples in that condition. The hierarchical clustering heatmap was produced using open-source R language (www.r-project.org) with Euclidean distance as distance measurement between clusters. The genes are arranged according to their Pearson correlation coefficient.

### Apoptosis Assays

PBMCs (10^7^/well) were incubated for 8 h in 24-well plates with the following reagents: 1) 2 µg/ml FLAG-tagged FasL crosslinked with 6 µg/ml of an anti-FLAG tag antibody, 2) 6 µg/ml anti-FLAG antibody alone, 3) 1.7 µg/ml trimeric gp160 HIV envelope protein (Protein Sciences), 4) 2×10^6^ CEM-IIIB cells [40], 5) 2×10^6^ CEM-IIIB cells plus 100 µg/ml T20 (Roche), 6) 2×10^6^ uninfected CEM cells (controls were performed using unrelated patient samples), or 7) media alone. After incubation, cells were collected and stained first with anti-BDCA-2-APC (Miltenyi) and anti-CD123-PE (BD Biosciences) followed by Annexin V-FITC (BD Biosciences) and 7-AAD (eBioscience). Samples were analyzed by FACSCanto (BD Biosicences).

### Statistical Analysis

Comparison of independent samples was done by the Kruskal-Wallis test with the Wilcoxon two-sample test. The Wilcoxon signed rank test was used to determine significance of paired data, and the adjustment of p values for multiple testing was done by the Bonferroni method.

## Supporting Information

Table S1 Microarray data. This spreadsheet lists all genes differentially expressed with a p-value<0.05 between pDCs from low- versus high-viremic individuals.
(0.14 MB CSV)
